# Study of craniofacial alterations and of the importance of the rapid maxillary expansion after tonsillectomy

**DOI:** 10.1590/S1808-86942012000200017

**Published:** 2015-10-20

**Authors:** Silvia Regina Amorim Pereira, Luc Louis Maurice Weckx, Cristina Lúcia Feijó Ortolani, Silvia Fuerte Bakor

**Affiliations:** a PhD in Sciences – UNIFESP-EPM (Adjunct Professor in Specialization Programs in Orthodontics); b Full Professor of Pediatric Otorhinolaryngology of the Department of Otorhinolaryngology and Head and Neck Surgery at the Medical School of the Federal University of São Paulo – UNIFESP – EPM (Head of the ENT-HNS Department of the UNIFESP – EPM); c PhD in Orthodontics – USP (Full Professor of Orthodontics – UNIP); d PhD in Sciences – UNIFESP – EPM (Researcher and Professor in Specialization Programs in Orthodontics)

**Keywords:** maxillofacial development, mouth breathing, palatal expansion technique, tonsillectomy

## Abstract

Obstructive hypertrophy of the tonsils and/or adenoids is associated with mouth breathing and can lead to facial imbalances. Adenotonsillectomy is not enough to treat the anatomic changes. Facial orthopedic techniques aid in morphological and functional recovery. This prospective longitudinal clinical study aimed to observe craniofacial changes after adenotonsillectomy and to verify the importance of linking rapid maxillary expansion to treatment.

**Method:**

Fifty-three children of both genders, aged 6 to 12 years, were allocated to: Group 1, 20 children with nasal breathing; and group 2, 33 children with obstructive hypertrophy of pharyngeal and/or palate undergoing adenotonsillectomy. After surgery, this group was subdivided into Group 2A, 16 patients not treated with rapid maxillary expansion; and Group 2B, 17 patients treated with maxillary rapid expansion. Frontal and lateral cephalometric measurements were made prior to surgery and after 14 months. Statistical analysis used the Kruskal-Wallis and Wilcoxon tests – significance level of 5%.

**Results:**

Adenotonsillectomy balanced transversal, sagittal and vertical growth in both groups, and was more effective in the group undergoing combined treatment.

**Conclusions:**

Adenotonsillectomy improved the facial growth of children with obstructive hypertrophy, which was more evident when associated with rapid maxillary expansion.

## INTRODUCTION

The obstructive hypertrophy of the palatine and pharyngeal tonsils is associated with oral breathing and, when it happens at the stage of facial growth, it may cause important morphofunctional unbalance. The most frequent morphological characteristics in mouth breathing patients are well known: long and narrow face, labial incompetence, retrognathic mandible and maxilla, narrow and deep upper arch, and a lower tongue rest position. Linder-Aronson reported that adenotonsillectomy alone is not enough when other anatomical changes are present, since they impair breathing and prevent growth from happening in a balanced way^1^, ^2^, ^3^.

The facial orthopedic techniques used to correct maxillary deformities help reestablish shape and function. The fast maxilla expansion (FME) is a technique based on the cleavage of the median palatine suture by means of a screw which pushes the hemi-maxillae apart^4^, ^5^, ^6^, ^7^.

Having in mind the very limitations of adenotonsillectomy in correcting anatomical and functional facial changes, it is known that the oral breathing child upon development must be treated by a multidisciplinary team. The goal of the present study is to consider growth changes in oral breathing children after adenotonsillectomy and study the importance of the fast maxilla expansion as part of the treatment.

## MATERIALS AND METHODS

The study was assessed and approved by the Ethics in Research Committee of the teaching institution where it was developed (report number 0427/04), and those responsible for the subjects in this study signed the Informed Consent Form.

### Sample

Made up by 53 children in the Pediatric Otorhinolaryngology Ward of the institution where it was conducted. Subjects between 6 and 12 years of age, from both genders, were broken down into:

*Group 1 or Control:* made up by 20 children, nine boys and 11 girls, with oral breathing proven by nasal endoscopy carried out in our institution, with a non–hypertrophic and non-obstructive pharyngeal tonsil occupying less than 40% of the choanal space^8^ and palatine tonsils level 0, +1 or level +2^9^. We took off the study those children who had had a past of orthodontic treatment and/or speech therapy, upper airways surgery and craniofacial malformation.
*Group 2 or Oral:* made up of 33 children with a nasal endoscopy diagnosis of obstructive hypertrophy of the pharyngeal tonsil (occupying 70% of the choanal space)^8^ and grades 3 or 4 of palatine tonsil hypertrophy (50 to 75% of air passage obstruction in the oropharynx, or more than 75% of air passage obstruction in the oropharynx)^9^. We took off the study those children who had craniofacial malformation; submitted to orthodontic treatment and/or speech therapy; prior surgery of the upper airways; persistent rhinitis and/or structurally modified concha; nasal septum deviation or any other upper airway obstacle that is not palatine and pharyngeal tonsil hypertrophy. After the diagnosis, all the patients in this group were submitted to adenotonsillectomy, by indication of the otorhinolaryngologist. After surgery, the group was randomly broken down into two subgroups.
*Subgroup 2A:* 16 children, six girls and 10 boys, not submitted to orthodontic treatment.
*Subgroup 2B:* 17 children, nine girls and eight boys, submitted to the Hyrax expansion device, installed 30 days after surgery. Initial activation was one complete turn (1.0mm) and during the 12 consecutive days it was one quarter turn in the morning and one quarter turn at night (0.5mm/day)^10^. The device was kept in place during four months^10^, ^11^ all the way to the bone new growth at the median palatine suture, when the device was removed and a removable palatine retention plate was installed; left in place for eight months, making up a total of 14 months of retention^11^.

### Cephalometric analysis

**Figure 1:**
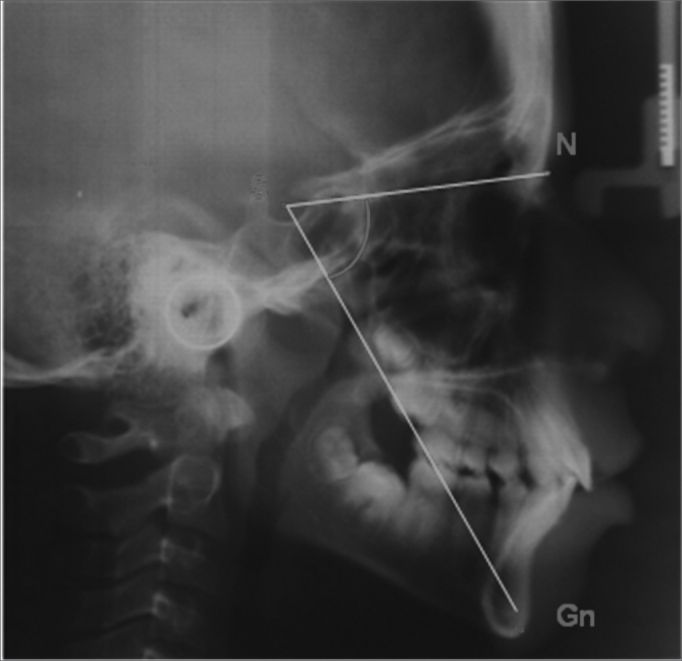
SN.Gn – Angle between the Saddle, Nasion and Gnatio points.
  • SN.GoMe: angle formed between the anterior skull base (SN) and the mandibular plane (GoMe), it establishes the vertical plane of the facial growth – represented by Figure 2.

Measures obtained by means of cephalometric radiographic measures in frontal and side views. Group 1 subjects were radiographed within a 14 month interval, and Group 2 subjects (a and b subgroups) were radiographed before and 14 months after surgery. The following measures were carried out:

*Side view*
  • SN.Gn (Y growth axis): angle formed between the Saddle, Nasionn and Gnatio points, it establishes the vertical plane of facial growth – represented by Figure 1.

**Figure 2:**
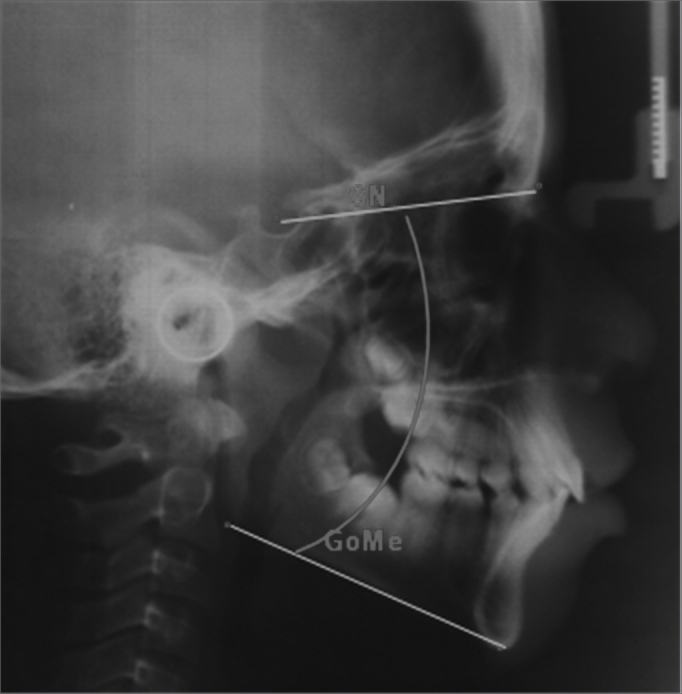
SN.GoMe – Angle between the anterior skull base (SN) and the mandibular plane (GoMe).
  • FMA: angle formed between the Frankfurt plane and the mandibular plane (GoMe), it establishes the vertical growth plane – represented by Figure 3.

**Figure 3:**
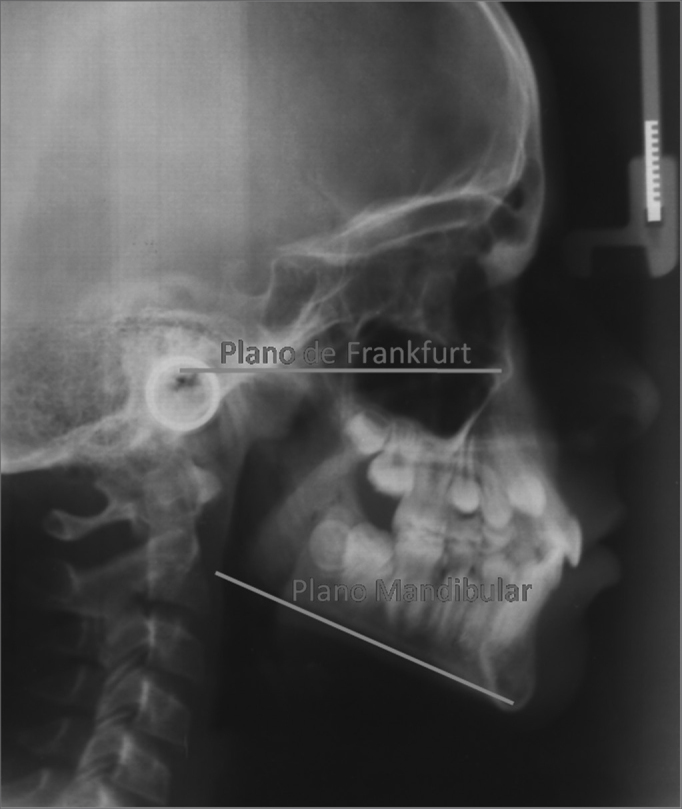
FMA – Angle between the Frankfurt plane and the mandibular plane.
  • Co-A: linear distance between the condyle and point A, it establishes the effective length of the maxilla – represented by Figure 4.

**Figure 4:**
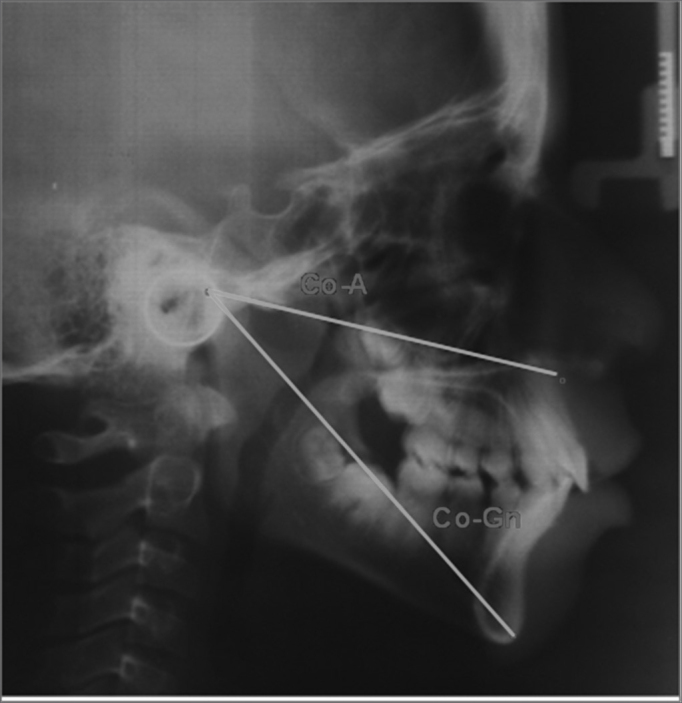
CoA e CoGn – CoA, linear distance from the condile point all the way to point A; CoGn, linear distance from the condyle point all the way to the Gnatio point.
  • Co-Gn: linear distance between the condyle point all the way to the Gnatio point, it establishes the effective length of the mandible – represented by Figure 4.
  • Nperp: linear distance from the point A all the way to the perpendicular Nasion line, it positions the maxilla in relation to N – represented by Figure 5.

**Figure 5:**
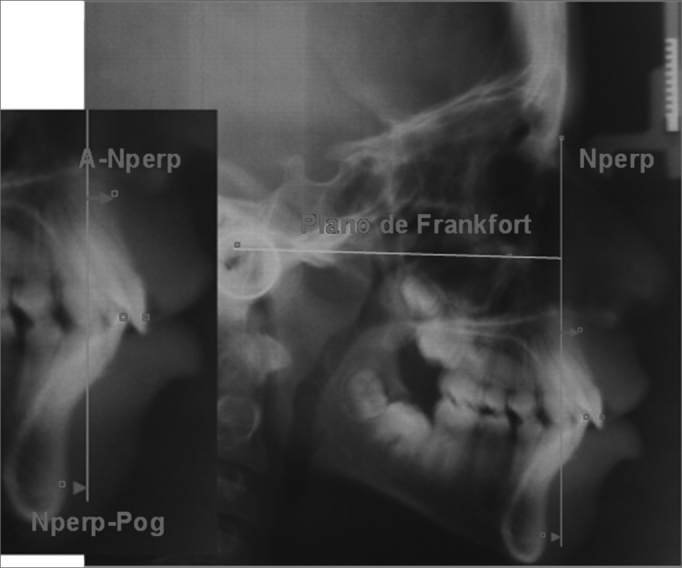
NperpA and NperpPg – NperpA, linear point from point A to the perpendicular Nasion line; NperpPg, linear distance from the Pog point all the way to the perpendicular Nasion line.
  • Nperp-Pg: linear distance from the Pog point all the way to the perpendicular Nasion line, it positions the mandible in relation to N – represented by Figure 5.
*Frontal view*
  • Maxilla width: cross-sectional linear measure of JL – JR – represented by Figure 6.

**Figure 6:**
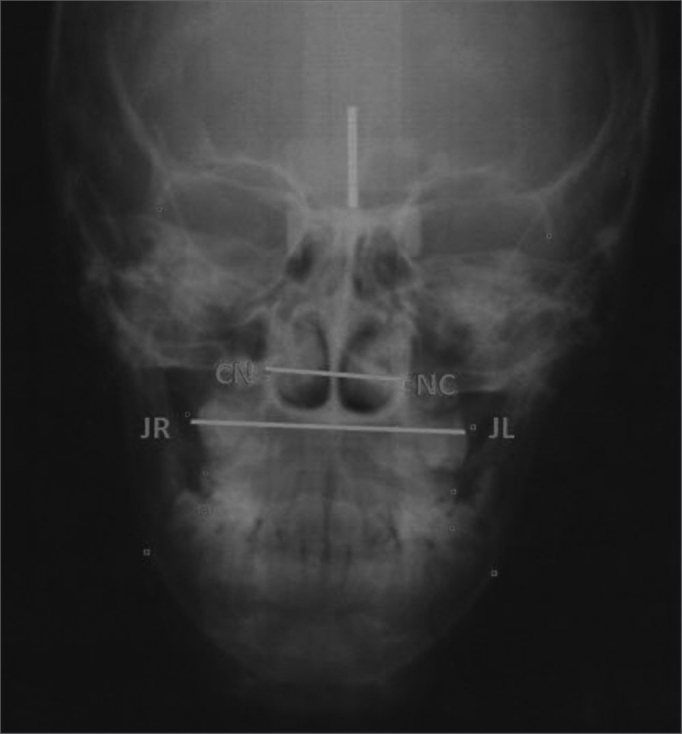
Nasal and maxillary widths – maxillary width: cross-sectional linear measure from JL to JR; nasal width, cross-sectional linear measure from CN to NC.
  • Nasal width: cross-sectional linear measure of CN – NC – represented by Figure 6.

### Statistical analysis

The Kolmogorov-Smirnov test was employed in order to study the sample symmetry, which did not follow the Gauss curve. In order to assess possible differences between the initial measures of the control group and oral subgroups, we used the non-parametric test for correlated measures from Kruskal-Wallis. For the possible differences among the measures before and after the procedures within each subgroup, we used the Wilcoxon non-parametric test for correlate measures. For the differences between the groups, for each variable, we used the Kruskal-Wallis non-parametric test for independent groups, complemented by the multiple comparisons test (Δ%), which measures the percentage variation and checks to see which of the groups had the most pronounced improvement.

## RESULTS

Table 1 shows the average values of the cephalometric variables obtained at the beginning of the study, in the three groups. For the side view variables, there were statistically significant differences in values SN.Gn, SN.GoMe, FMA and Nper-Po; and there were no differences in Co-A, Co-Gn and Nperp-A; for the frontal view variables, there was a statistically significant difference in the maxilla width value and there were no differences in nasal width.

**Table 1:** Intergroup mean values of the INITIAL variables.

Measures	G 1	G 2 A	G 2 B	Kruskal-Wallis
	n=20	n = 16	n = 17	*p*
		Side view		

Sn.Gn (degrees)	67.2	71.2	71.8	0.002
Sn.GoMe (degrees)	33.6	40.2	40.8	0.001
FMA (degrees)	25.2	29.8	30.2	0.001
Co-A (mm)	85	83.3	85.4	0.343
Co-Gn (mm)	109.3	108	110	0.386
Nperp-A (mm)	1.8	1.1	1.3	0.726
Nperp-Po (mm)	−2.3	−5.9	−6.5	0.020

		Frontal view (mm)		

Nasal width (mm)	28.8	27	27.9	0.231
Maxillary width (mm)	63.2	58.1	59.9	0.000

Table 2 compares the control group variables at the beginning and after 14 months of cephalometric follow up. We found significant differences in the following variables: Co-A, Co-Gn, Nperp-A, maxilla width and nasal width.

**Table 2:** INITIAL X FINAL Mean values of the control group (n = 20).

Measures	Moment	Mean	Wilcoxon *p*
Side view			

SN.Gn (graus)	Initial	67.2	0.446
	Final	66.8	

SN.GoMe (graus)	Initial	33.6	0.480
	Final	33.4	

FMA (graus)	Initial	25.2	0.216
	Final	24.8	

Co-A (mm)	Initial	85	0.000
	Final	88	

Co-Gn (mm)	Initial	109.3	0.000
	Final	112.4	

Nperp-A (mm)	Initial	1.8	0.035
	Final	2.9	

Nperp-Po (mm)	Initial	−2.3	0.274
	Final	−1.4	

Frontal view (mm)			

Nasal Width (mm)	Initial	28.8	0.000
	Final	30.2	

Largura Maxilar (mm)	Initial	63.2	0.000
	Final	65.2	

Table 3 compares the oral 2 A subgroup variables, which were submitted to adenotonsillectomy, without orthopedic intervention. The measures were obtained before and after surgery, within a 14-month time interval. There was a statistically significant difference in the following measures: Sn.GoMe, Co-A and Co-Gn, and there were no statistically significant differences in measures: FMA, SN.Gn, Nperp-Po and Nperp-A. The frontal view cephalometric measures showed statistically significant differences on nasal width and maxilla width.

**Table 3:** INITIAL X FINAL mean values from Group 2 A (n = 16).

Measures	Moment	Mean	Wilcoxon *p*
Lateral view			

SN.Gn (degrees)	Initial	71.2	0.152
	Final	70.4	

SN.GoMe (degrees)	Initial	40.2	0.022
	Final	39	

FMA (degrees)	Initial	29.8	0.095
	Final	28.2	

Co-A (mm)	Initial	83.3	0.006
	Final	85.1	

Co-Gn (mm)	Initial	108	0.000
	Final	111.9	

Nperp-A (mm)	Initial	1.1	0.956
	Final	1.3	

Nperp-Po (mm)	Initial	−5.9	0.127
	Final	−4.5	

Frontal view (mm)			

Nasal width (mm)	Initial	27	0.006
	Final	28	

Maxillary Width (mm)	Initial	58.1	0.000
	Final	61.3	

Table 4 compares the variables from subgroup oral 2 B, which was submitted to adenotonsillectomy, followed by the orthopedic maxilla expansion approach. The results showed that there was a statistically significant differences in measures SN.Gn, Co-A and Co-Gn and there were no statistically significant difference in measures FMA, SN.GoMe, Nperp-A and Nperp-Po. In the frontal view we found a statistically significant difference in the nasal width and maxilla width measures.

**Table 4:** INITIAL X FINAL mean values from Group 2 B (n = 17).

Measures	Moment	Mean	Wilcoxon *p*
Lateral view			

SN.Gn (degrees)	Initial	71.8	0.042
	Final	70.9	

SN.GoMe (degrees)	Initial	40.8	0.093
	Final	39.6	

FMA (degrees)	Initial	30.2	0.187
	Final	29.3	

Co-A (mm)	Initial	85.4	0.019
	Final	86.8	

Co-Gn (mm)	Initial	110	0.000
	Final	114	

Nperp-A (mm)	Initial	1.3	0.642
	Final	1	

Nperp-Po (mm)	Initial	−6.5	0.93
	Final	−4.8	

Frontal view (mm)			

Nasal width (mm)	Initial	27.9	0.001
	Final	29.4	

Maxilarry width (mm)	Initial	59.9	0.006
	Final	61.6	

Table 5 compares the mean intergroup values of all the variables studied. There was a statistically significant difference in variable Co-A among the oral subgroups which were not submitted to ERM and those who were submitted to ERM. The variables: CoGn, Nperp-Po, Sn.Gn, SnGoMe, Nperp-A and FMA did not show statistically significant differences. In the frontal view, there was a statistically significant difference in the nasal width and maxilla width variables.

**Table 5:** Intergroup mean values of the FINAL variables.

Measures	G 1 n=20	G 2 A n=16	G 2 B n=17	Kruskal-Wallis *p*
		Side view		

Sn.Gn (degrees)	−0.5	−1	−1.3	0.542

Sn.GoMe (degrees)	−0.4	−3.3	−2.7	0.234

FMA (degrees)	−1.6	−5.4	−2.6	0.631

Co-A (mm)	3.5	2.2	1.7	0.032

Co-Gn (mm)	2.8	3.6	3.7	0.371

Nperp-A (mm)	82.4	21.7	−32.6	0.196

NPerp-Po (mm)	69.9	−20.4	17	0.858

		Frontal view		

Nasal width (mm)	5	4.5	8.2	0.013

Maxillary width (mm)	2.8	2.7	6.9	0.000

## DISCUSSION

The variables which express the facial growth pattern on the vertical direction (SN.Gn, SN.GoMe, FMA) had statistically significant differences when the control group was compared to the oral subgroups studied (Table 1), which means that mouth breathers had a vertical growth pattern vis-à-vis the control group before being submitted to surgery. This growth pattern seems to be a consensus among numerous authors in the literature^1^, ^12^, ^13^, ^14^, ^15^, ^16^, ^17^, ^18^, ^19^, ^20^, ^21^, ^22^, ^23^, ^24^, ^25^. The Nper-Po variable studies the sagittal proportions of the mandible and we noticed a significant retropositioning of the mandible in the oral groups upon study onset (Table 1), in agreement with the publications available^1^, ^12^, ^13^, ^14^, ^15^, ^16^, ^17^, ^18^, ^19^, ^20^, ^21^, ^22^, ^23^, ^24^, ^25^, ^26^. For the frontal view variables, there were statistically significant differences insofar as the maxillary width is concerned, which stresses the maxillary atresia of the oral subgroups at the onset of the study. (Table 1). The mouth breathing children had maxillary atresia before the adenotonsillectomy, seen upon the frontal view cephalometric radiography, and this aspect is also in agreement with the related studies^1^, ^22^, ^27^.

As we compare the control group variables individually upon onset and after 14 months of cephalometric follow up, we found significant differences in variables: Co-A, Co-Gn and Nperp-A, maxillary and nasal widths. These results mean that the children in the study had the expected sagittal and cross-sectional facial growth, with a stability of the cross-sectional measures (Table 2).

As we studied the oral 2 A subgroup (Table 3), which was submitted only to adenotonsillectomy, without orthopedic intervention, we noticed a significant difference in the Sn.GoMe value, which shows that the vertical growth was controlled after surgery. The significant variables Co-A and Co-Gn indicate maxillomandibular sagittal growth, which occurred similarly to the control group, with indications of facial profile balance. In the frontal view, we found statistically significant differences in the nasal and maxillary widths, a very favorable result, since it points to maxillary atresia control.

As we compared the variables from subgroup oral 2B (Table 4), which was submitted to surgical treatment together with orthopedic intervention, we noticed that the vertical growth pattern was controlled (significant SN.Gn), and the maxillomandibular sagittal measures Co-A and Co-Gn were significantly changed, with a consequent improvement in facial profile. In the frontal view, we found a significant difference in maxillary width and nasal width measures, a cross-sectional gain sign – a very important aspect in reestablishing dental arch perimeters.

The intergroup analysis depicted on Table 5 compares the mean values from all the variables studied. Both oral subgroups had control over the vertical trend of growth seen upon the onset of the study. We found a statistically significant difference in the Co-A variable among the oral subgroups, indicating that the maxillary growth in the sagittal direction (Co-A) of the children submitted to adenotonsillectomy and treated with maxillary expansion was statistically lower when compared to the children submitted to surgery. This difference happened because of the expected orthopedic variations in remodeling the maxillary sagittal growth caused by the expansion device^11^. Considering the convex profile frequently seen in oral breathers, we understand that the ERM result showed an advantage for introducing efficient maxillary sagittal growth control. In the frontal view, the group treated in association with ERM was also significantly benefited by the cross-sectional gain. Studies published in the literature found nasal atresia in their series of oral breathers and reported that the device causes maxilla expansion and it also increases nasal cavity volume, which improves airflow and reduces oral breathing influence over skeletal structures^4^, ^5^, ^6^.

The most evidently modified cross-sectional and sagittal variables after treatment associated to ERM tend to provide more stability and balance to the remaining facial growth.

## CONCLUSIONS

Adenotonsillectomy balanced the facial vertical growth of patients with oral breathing in this study, even in the untreated group with fast maxilla expansion, and the bone remodeling adjusts induced by facial orthopedics in the cross-sectional and sagittal directions were more evident.
